# A miR-335/COX-2/PTEN axis regulates the secretory phenotype of senescent cancer-associated fibroblasts

**DOI:** 10.18632/aging.100987

**Published:** 2016-06-29

**Authors:** Tasnuva D. Kabir, Ross J. Leigh, Hataitip Tasena, Massimiliano Mellone, Ricardo D. Coletta, Eric K. Parkinson, Stephen S. Prime, Gareth J. Thomas, Ian C. Paterson, Donghui Zhou, John McCall, Paul M. Speight, Daniel W. Lambert

**Affiliations:** ^1^ Integrated Biosciences, School of Clinical Dentistry, University of Sheffield, S10 2TA, UK; ^2^ Department of Surgical Sciences, Dunedin Medical School, Dunedin, University of Otago, Dunedin Hospital, Dunedin 9016, New Zealand; ^3^ Faculty of Medicine Cancer Sciences Unit, Southampton University, Somers Building, Southampton SO16 6YD, UK; ^4^ Department of Oral Diagnosis, School of Dentistry, University of Campinas, Piracicaba-SP, Brazil; ^5^ Centre for Clinical & Diagnostic Oral Sciences, Institute of Dentistry, Barts and the London School of Medicine and Dentistry, Queen Mary University of London, London E1 2AD, UK; ^6^ Department of Oral and Craniofacial Sciences, and Oral Cancer Research and Coordinating Centre, Faculty of Dentistry, University of Malaya, Malaya, Malaysia; ^7^ Department of Biochemistry, School of Medical Sciences, University of Otago, Dunedin 9054, New Zealand

**Keywords:** miR-335, PTEN, fibroblast, CAF, SASP, COX-2

## Abstract

Senescent cancer-associated fibroblasts (CAF) develop a senescence-associated secretory phenotype (SASP) that is believed to contribute to cancer progression. The mechanisms underlying SASP development are, however, poorly understood. Here we examined the functional role of microRNA in the development of the SASP in normal fibroblasts and CAF. We identified a microRNA, miR-335, up-regulated in the senescent normal fibroblasts and CAF and able to modulate the secretion of SASP factors and induce cancer cell motility in co-cultures, at least in part by suppressing the expression of phosphatase and tensin homologue (PTEN). Additionally, elevated levels of cyclo-oxygenase 2 (PTGS2; COX-2) and prostaglandin E2 (PGE2) secretion were observed in senescent fibroblasts, and inhibition of COX-2 by celecoxib reduced the expression of miR-335, restored PTEN expression and decreased the pro-tumourigenic effects of the SASP. Collectively these data demonstrate the existence of a novel miRNA/PTEN-regulated pathway modulating the inflammasome in senescent fibroblasts.

## INTRODUCTION

The tumour microenvironment plays a key role in cancer growth and metastasis. Whilst it is likely that the cells and extracellular matrix (the tumour stroma) underlying an epithelial malignancy are initially hostile to tumour infiltration, it is becoming clear that stromal adaptations take place in response to contextual cues which ultimately enable tumour dissemination [1]. The predominant cellular component of the tumour stroma, the fibroblast, undergoes complex and heterogeneous phenotypic alterations, often referred to collectively and non-specifically as ‘activation’ [2]. This term covers a range of cancer-associated fibroblast (CAF) phenotypes that are supportive of, and possibly hostile to, tumour invasion, the molecular aetiology of which is poorly understood.

A great deal of attention has been paid to the concept of α-smooth muscle actin positive ‘myofibroblastic’ CAF [3], but it has become clear that this is not the only CAF phenotype. Senescent CAF are also present in the tumour microenvironment [4] as well as in premalignant lesions [5-8] including those that precede the development of neoplasia in oral sub-mucosal fibrosis [8]. These senescent cells, characterized by having undergone irreversible growth arrest but remaining metabolically active, generate an enhanced and altered secretome termed the senescence-associated secretory phenotype (SASP) [9]. The SASP, which contains elevated levels of growth factors, cytokines, ECM components and other factors, contributes to the generation of a pro-inflammatory, pro-metastatic extracellular milieu [9]. During oncotherapy most anti-cancer drugs, such as cisplatin, induce either apoptosis or senescence of cancer cells to suppress tumour growth [10, 11] and this may also provoke senescence in the neighbouring stroma [12], with detrimental effects [13]. SASP mediated cross-talk between stroma and cancer cells may, therefore, be critical in determining the success and failure rates of chemotherapy and influence the development of chemotherapy resistance, disease recurrence or progression, and ultimately, patient survival.

Senescent CAF are likely to predominantly develop from resident fibroblasts in response to micro-environmental insults (such as reactive oxygen species generated by aberrant metabolic activity in cancer cells), as well as those derived from the environment (such as irradiation, chemotherapy and cigarette smoke) and natural aging [14]. The molecular mechanisms underlying the development of the resulting pro-tumourigenic SASP, however, remain poorly understood. Elucidating the mechanisms responsible for generating the SASP could have translational benefits since stromal fibroblasts are generally believed to be genetically stable [15] and therefore less vulnerable to developing resistance to therapy, a significant problem with the pharmacological targeting of genetically unstable tumour cells.

Here we show that the development of an inflammatory SASP by fibroblasts induced to senesce by cisplatin and other DNA damaging agents or aging, as well as in innately senescent CAF, is stimulated by miR-335 and regulated by COX-2 and PTEN. Collectively our data suggest that combined targeting of COX-2 and signaling downstream of PTEN, in association with conventional chemotherapy, may ameliorate the pro-tumourigenic effects of chemotherapy on the tumour microenvironment.

## RESULTS

### Cisplatin induces senescence in primary oral fibroblasts and provokes the development of a pro-tumourigenic SASP

**Figure 1:**
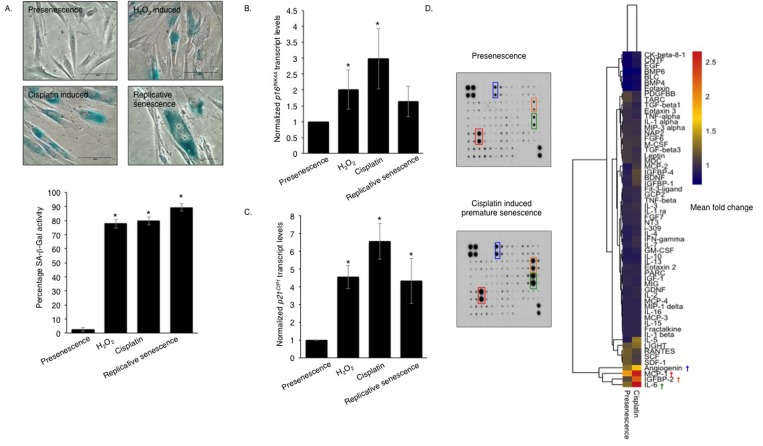
Genotoxic stress induces a pro-inflammatory SASP in normal human oral fibroblasts Senescence-associated β-galactosidase activity (**A**) was measured in human oral fibroblasts treated with hydrogen peroxide (H_2_O_2_) and cisplatin at day 15 post-treatment and in replicative senescent and untreated presenescent control cells (n=4). qRT-PCR showed senescent fibroblasts expressed more cyclin dependent kinase inhibitor (CDKI): p16^INK4A^ (**B**) and p21^CIP1^ (**C**) than presenescent controls (n=4). The heat-map demonstrates the cytokine expression profile of senescent fibroblasts measured by human cytokine antibody array (**D**) (n=2). All experiments were performed independently as indicated by n and with technical repeats. The bars represent mean ± STDEV (**B, C**) or mean ± SEM. *p<0.05, was determined by one-way ANOVA with post-hoc correction by Holm-Sidak method (**A**) and Dunn's method (**B,C**). (See Supplementary Figure S1).

**Figure 2:**
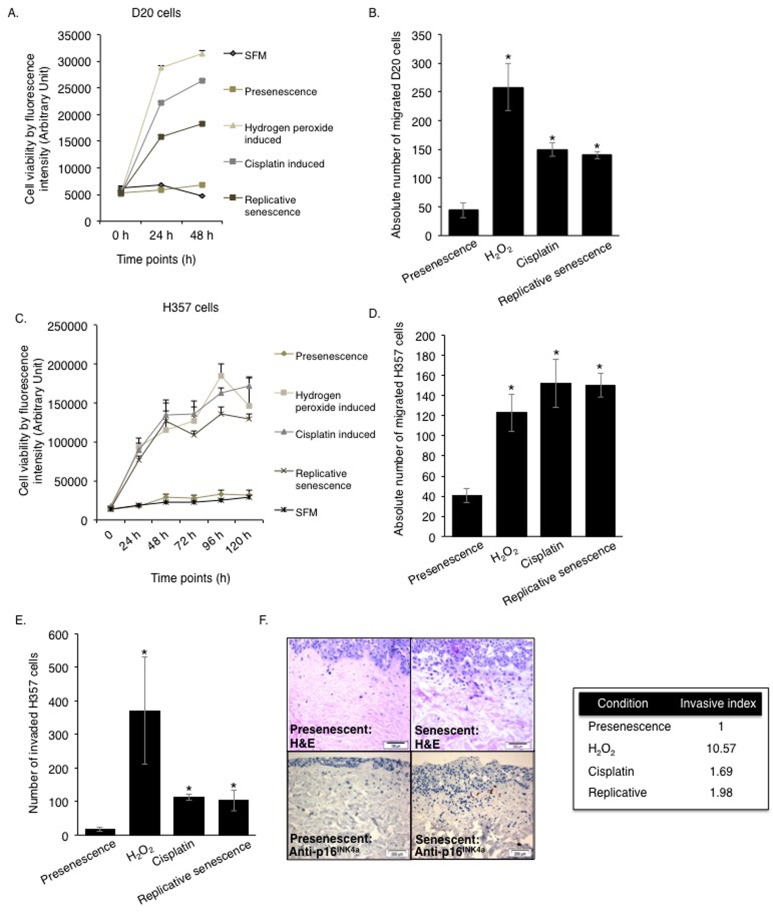
The SASP engenders a protumourigenic phenotype to the senescent oral fibroblasts Soluble factors secreted by senescent fibroblasts stimulated proliferation and migration of D20 (**A**-**B**) and H357 cell lines (**C**-**D**) in 2D assay (n=3). The invasiveness of H357 cells was also increased by senescent fibroblasts in both 2D and 3D organotypic models (**E**-**F**) (n=3). Immunohistochemistry for p16^INK4A^ was performed in paraffin embedded sections of 3D organotypic models to confirm presence of senescent fibroblasts (**F,** left lower panel). All experiments were performed independently as indicated by n and with technical repeats. The bars represent mean ± SEM. *p<0.05, was determined by one-way ANOVA with post-hoc correction by Tukey test (**B**) and Dunn's method **(D-E)**. Two-way repeated measure ANOVA was performed to determine statistical significance in proliferation assay (**A, C**) with post-hoc correction by Holm-Sidak method. (See Supplementary Figure S2).

Although it has been reported that primary human oral fibroblasts are able to develop a senescence-associated secretory phenotype (SASP), the constituents of this have not previously been characterized and the ability of chemotherapeutics to induce the SASP in these cells has not been explored. In order to address these questions, fibroblasts were exposed to DNA damaging agents (H_2_O_2_ and cisplatin), or cultured to replicative senescence and the composition of the resulting secretome was analysed. Successful induction of senescence was demonstrated by positive senescence-associated (SA) β-galactosidase staining (Fig 1A), elevated *p16^INK4A^* levels (Fig 1B) and increased levels of *p21^CIP1^* (Fig 1C). Analysis of conditioned media collected from prematurely senescent fibroblasts up to 15 days after induction of senescence revealed increased pro-matrix metalloproteinase 2 (pro-MMP2) activity (Fig S1A-C) and widespread alterations in the constituents of the SASP compared to proliferating controls (Fig 1D). Increased expression and secretion of IL-6 and MCP-1 in senescent cells were confirmed by qRT-PCR (except replicative senescence) and ELISA, respectively (Fig S1D-G). All secreted proteins were normalized to fibroblast cell density. Senescent fibroblasts also displayed increased α-SMA positive actin filaments compared to proliferating controls (Fig S1H). Conditioned media from senescent fibroblasts stimulated proliferation, chemotaxis and invasion of dysplastic (pre-malignant) keratinocytes and malignant epithelial cells (Fig 2A-E and Fig S2A-B). Incorporation of senescent fibroblasts into an organotypic model of the malignant oral mucosa [16] increased the invasive index of non-metastatic H357 [17] cancer cells (Fig 2F).

### Widespread changes in miRNA expression are associated with the development of the SASP

**Figure 3:**
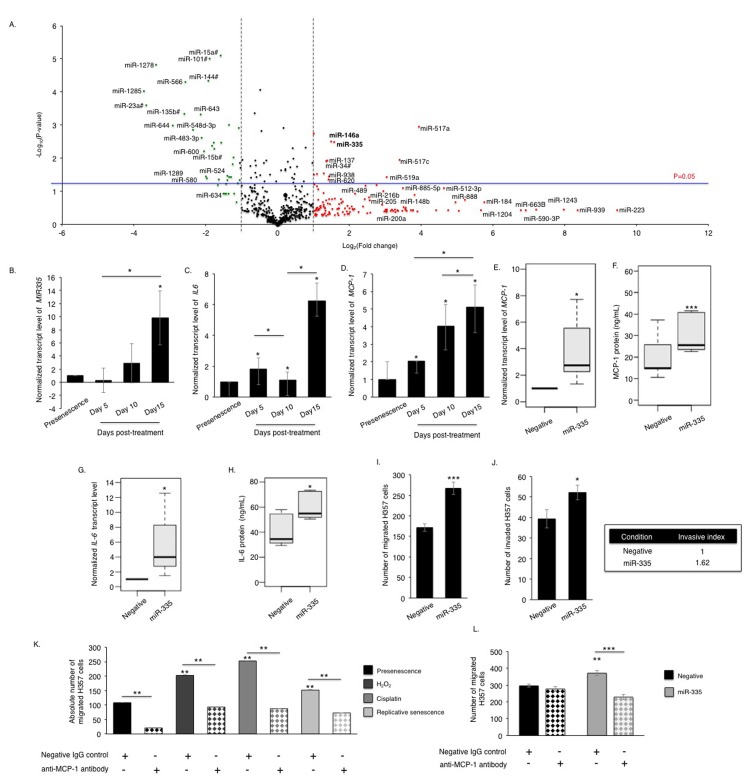
Senescent oral fibroblasts differentially express SASP-associated miRNAs The volcano plot illustrates the differentially expressed miRNA signature in cisplatin induced senescent oral fibroblasts. cDNA synthesized from RNA isolated from proliferating fibroblasts and fibroblasts induced to senesce using cisplatin (RNA isolated 15 days post-senescence) was analyzed using TaqMan miRNA tiling low density array (TLDA) to determine miRNA expression profile in senescent fibroblasts (n=3) (**A**). qRT-PCR showed that miR-335 (**B**) levels gradually increase in senescent fibroblasts with a time course corresponding to the increase in the levels of SASP components IL-6 (**C**) and MCP-1 (**D**), (n=3). Over-expression of miR-335 in young fibroblasts increased synthesis and secretion of MCP-1 (**E**-**F**) and IL-6 (**G**-**H**) (n=3), and stimulated chemotaxis and invasion of H357 cells in 2D-assay (n=3) (**I-J**). Blockade of MCP-1 in conditioned media of senescent fibroblasts significantly reduced migration of H357 cells compared to negative isotype IgG treated control (n=3) (**K**). Blockade of MCP-1 in conditioned media derived from miR-335 over-expressing fibroblasts also reduced H357 cell migration than those fibroblasts transfected with negative miRNA control (n=3) (**L**). All experiments were performed independently as indicated by n and with technical repeats. The bars represent mean ± STDEV (**B**-**E**, **G**) or mean ± SEM (**F**, **H**-**L**). *p<0.05 was determined by paired student's t-test (**A**, **E**, **I**-**L**), two-way repeated measure ANOVA for time course studies with post-hoc corrections by Holm-Sidak method (**B**-**D**), and Mann-Whitney U-test (**F**,**H**). (See Supplementary Figure S3 and Supplementary Table S1).

Profiling of cDNA synthesized from RNA extracted from senescent fibroblasts, 15 days after induction of senescence with cisplatin, revealed widespread changes in miRNA expression levels (Fig 3A and Table S1), including elevated miR-146a, a microRNA previously reported to be associated with secretion of inflammatory cytokines by senescent fibroblasts [18]. Following validation of changes in candidate miRNA expression in a number of cultures, and assessment of expression changes in fibroblasts induced to senesce by different stimuli (H_2_O_2_ and replicative exhaustion) by qPCR, miR-335-5p (thenceforth referred to as miR-335) was selected for further analysis (Fig S3A-C). Elevated levels of miR-335 were previously reported in aged human tissues and found conserved across species [19, 20]. Its over-expression induced premature ageing in human sarcoma cell lines and mesenchymal stem cells [21, 22]. We observed that miR-335 was significantly up-regulated in senescent fibroblasts irrespective of the method of senescence induction, and the time course of induction of miR-335 was in accordance to that of IL-6, MCP-1 and increased catalytic activity of MMP-2 (Fig 3B-D, Fig S1A), suggesting that miR-335 was a putative candidate as a regulator of SASP development. Accordingly, heterologous over-expression of miR-335 increased the expression and secretion of a panel of SASP markers including MCP-1 (rho=0.75, p-value=0.03), IL-6 (rho=0.95, p-value=0.0002) and MMP-2 (rho=0.7, p-value=0.035) (Fig 3E-H, Fig S3D). Over-expression of miR-335 in fibroblasts increased migration and invasion of malignant H357 cells in indirect co-culture (Fig 3I-J). No effect was observed on the acquisition of senescence assessed by the levels of *p21^CIP1^* and *p16^INK4a^* (Fig S3E-F) and negative SA-β-Gal activity (data not shown), consistent with previous reports by Campisi and colleagues that senescence and the development of SASP are two independent events [14, 23]. As stromal MCP-1 has recently been reported to stimulate chemotaxis of oral cancer cells [24, 25], we examined the effects of functionally blocking MCP-1 in miR-335 overexpressing fibroblasts on the migration and invasion of cancer cells. Blockade of secreted MCP-1 in conditioned media from senescent fibroblasts significantly attenuated the migration and invasion of H357 cells (Fig 3K, Fig S3G). Blockade of secreted MCP-1 in conditioned media of proliferating fibroblasts also produced a profound reduction in migration and invasion of H357 cells in co-culture, suggesting MCP-1 also plays a role in communication between proliferating fibroblasts and cancer cells. Alternatively, it is also plausible that within the proliferating population there exists a subset of MCP-1 secreting senescent fibroblasts, which stimulate cancer cell migration. Inhibition of MCP-1 in conditioned media of oral fibroblasts over-expressing miR-335 significantly reduced chemotaxis of H357 cells compared to control cells transfected with a non-targeting synthetic miRNA (Fig 3L).

### miR-335 targets PTEN to sustain elements of the SASP

**Figure 4:**
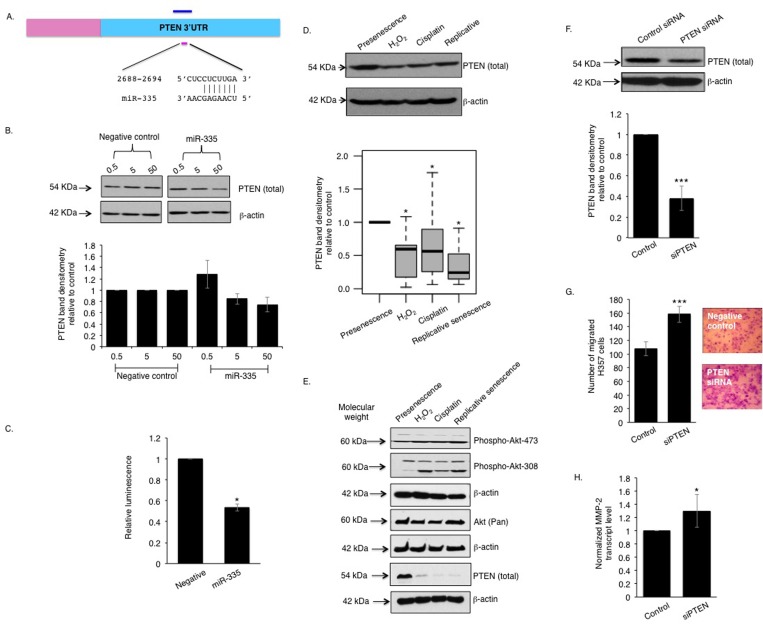
miR-335 represses PTEN function in senescent oral fibroblasts The 3′UTR of PTEN bears a conserved seed sequence complementary to miR-335 (**A**). Over-expression of miR-335 in oral fibroblasts negatively regulated PTEN protein level in dose-response manner (n=3) (**B**). Co-transfection of miR-335 with pmiR-reporter vector bearing a 1.45 kb insert of PTEN 3′UTR showed diminished luminescence compared to controls (n=3) (**C**). Western blot showed PTEN expression is reduced in senescent fibroblasts, compared to presenescence (proliferating) controls (n=9) (**D**) and this is associated with an increased phosphorylation of Akt (**E**). Transient knockdown of PTEN in oral fibroblasts stimulated transmigration of H357 cells *in vitro* (n=3) and increased MMP2 transcript levels (**F**-**H**). All experiments were performed independently as indicated by n and with technical repeats. The bars represent mean ± SEM or mean ± STDEV (**H**). *p<0.05 and ***p<0.01, were determined by paired student's t-test (**C**, **F**-**H**), Mann-Whitney U-test (**D**). The arrows indicate the respective molecular weight of detectable proteins in western blot using EZ-Run prestained rec protein ladder. (See Supplementary Figure S4 and Supplementary Table S2-S3).

*In silico* pathway analysis revealed that the phosphoinositide 3-kinase (PI3-K) and Akt signaling pathway ranked highest amongst fifty other pathways and the genes interacting in this pathway are predicted to be targeted by twenty-eight of the differentially expressed miRNAs including miR-335 (Table S2, Fig S4A-B). PTEN, a regulator of PI3-K and Akt signalling previously reported to be involved in stromal-tumour interactions and senescence [26, 27], is a putative protein-coding target of miR-335, and of a number of other miRNAs identified as up-regulated in senescent cells displaying a robust SASP (Fig 4A, Table S3). Over-expression of miR-335 caused a dose-dependent decrease in PTEN protein levels in primary oral fibroblasts (Fig 4B) and suppressed luciferase expression from a construct containing the firefly luciferase coding region directly 5′ upstream of a fragment of the PTEN 3′UTR containing the putative binding sites for miR-335 (Fig 4C). Analysis of PTEN expression in senescent fibroblasts revealed a significant decrease compared to proliferating cells irrespective of the method of senescence induction (Fig 4D). This reduction was independently confirmed by RNA-seq analysis of transcriptomic changes in senescent fibroblasts compared to proliferating controls (Mellone et al, manuscript in submission). Increased Akt phosphorylation (indicative of reduced PTEN function) was also observed in senescent fibroblasts (Fig 4E). Transient knockdown of endogenous PTEN using siRNA enhanced the capacity of normal oral fibroblasts to stimulate chemotaxis of malignant cells *in vitro* (Fig 4F-G) accompanied by a significant increase in secretion of the SASP marker MMP-2 (Fig 4H) and a trend towards increasing IL-6 levels (p=0.360) (Fig S4C).

### COX-2 signalling promotes miRNA-mediated PTEN reduction and drives the development of the SASP

**Figure 5:**
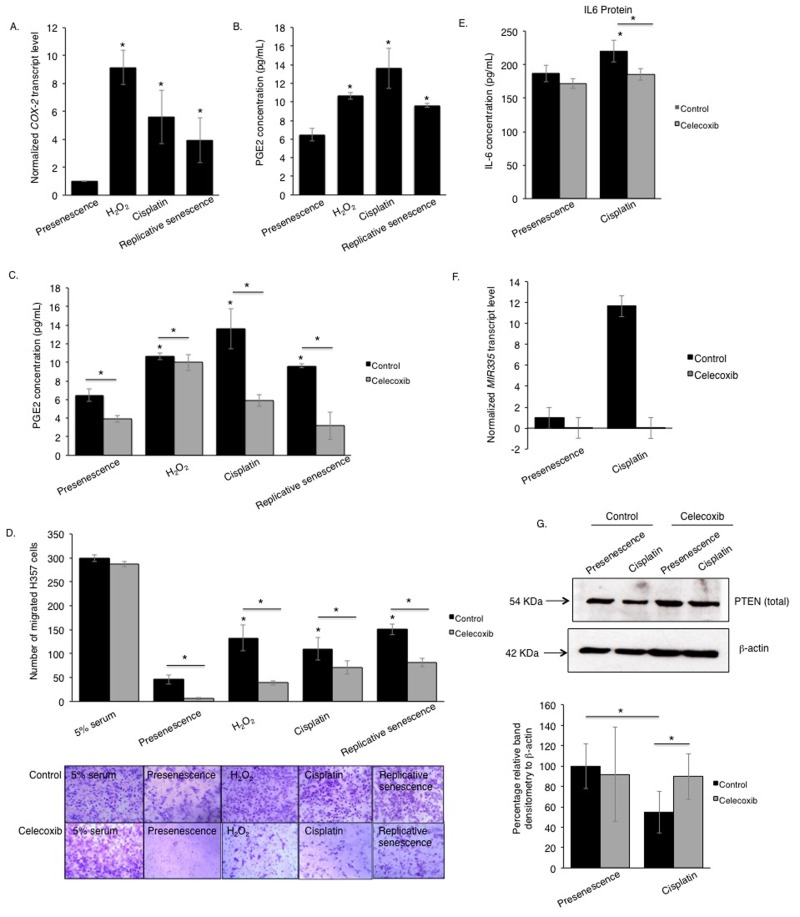
The pro-tumourigenic SASP of human senescent oral fibroblasts depends on elevated secretion of PGE2 qRT-PCR showed increased expression of COX-2 mRNA in senescent oral fibroblasts compared to proliferating controls (n=3) (**A**). Senescent oral fibroblasts secreted more PGE2 than proliferating control (n=3) (**B**). Treatment of senescent and control oral fibroblasts with celecoxib, a selective COX-2 inhibitor, diminished PGE2 secretion (n=3) (**C**). Blockade of COX-2 activity in both senescent and proliferating control oral fibroblasts dramatically attenuated the migration of H357 cells towards fibroblast derived conditioned medium (n=3) (**D**). Celecoxib treated fibroblasts secreted less IL-6 into the conditioned media (both senescent and proliferating) (n=3) (**E**). qRT-PCR and western blot showed COX-2 inhibition in senescent fibroblasts is associated with declined transcript levels of SASP associated miRNAs: miR-335 (**F**) (n=2) and increased expression of PTEN protein (**G**), respectively (n=4). All experiments were performed independently as indicated by n and with technical repeats. The bars represent mean ± STDEV (**A**, **F**) or mean ± SEM. *p<0.05, by paired student's t-test (**G**), one-way ANOVA with post-hoc correction by Dunn's method (**A**) and Holm-Sidak method (**B**) and two-way ANOVA for determining functional activity of COX-2 with post-hoc correction by Holm-Sidak method (**C**) and Bonferroni t-test (**D**-**E**). (See Supplementary Figure S5 and Supplementary Table S1).

Elevated prostaglandin (PGE2) production by COX-2 is known to be a feature of senescent cells [28], and recombinant PGE2 can induce senescence in fibroblasts [28-30]. COX-2-regulation of the PTEN/Akt signalling is well documented in osteoblasts [31] but its role in fibroblasts is less well understood [32, 33]. The beneficial effects of using nonselective cyclooxygenase inhibitors in combination with chemotherapy [34] compelled us to examine whether COX-2 activity played a role in modulating the SASP in primary oral fibroblasts. We analyzed the levels of COX-2 transcript and the secreted product of its catalytic activity, PGE2, in fibroblasts induced to senesce by cisplatin or H_2_O_2_, and in replicative senescent cells. COX-2 transcripts were higher in senescent fibroblasts than proliferating controls and this was associated with elevated levels of secreted PGE2 in conditioned media isolated from senescent cells (Fig 5A-B). IL-6 is characterized as a COX-2-dependent cytokine necessary to activate numerous oncogenic signals acting downstream of COX-2 in cancer cells via activating STAT3 (signal transducer and activator of transcription 3) [35]. Selective inhibition of COX-2 activity with celecoxib in senescent fibroblasts significantly constrained the release of PGE2 and SASP component IL-6, and diminished chemotaxis of cancer cells *in vitro* to levels similar to those of untreated proliferating control (Fig 5C-E). Celecoxib also inhibited cancer cell migration towards conditioned media of proliferating fibroblasts to a greater extent than its senescent counterparts indicating the presence of other COX-2 and IL-6 independent SASP components in the conditioned media of senescent fibroblasts (Fig 5D, Fig S5A-B). Celecoxib neither induced senescence in proliferating fibroblasts nor altered SA β-activity of senescent fibroblasts (Fig S5C). In contrast celecoxib reduced expression of miR-335 in senescent fibroblasts compared to proliferating controls and this was associated with restoration of PTEN expression (Fig 5F-G).

### A COX-2 stimulated miR-335/PTEN regulated SASP exists in senescent cancer-associated fibroblasts

**Figure 6:**
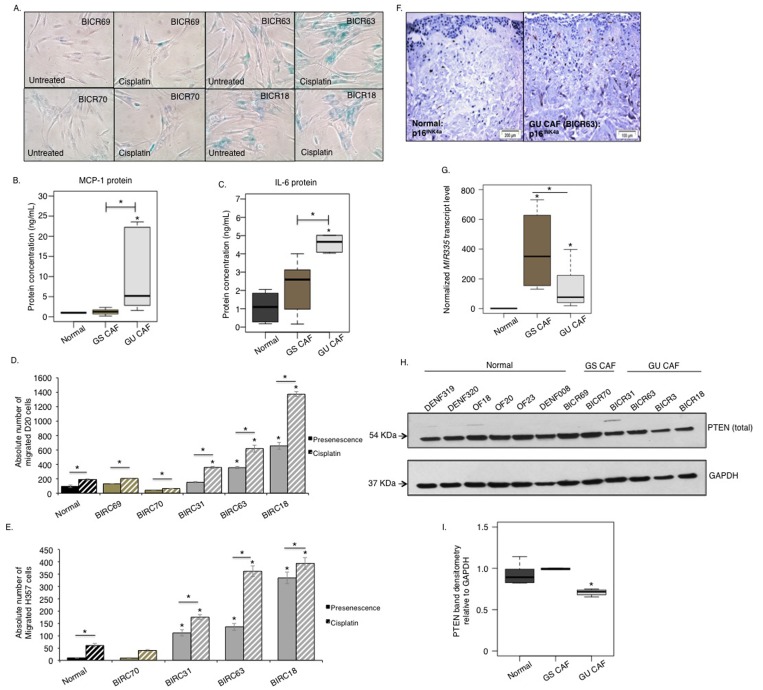
A miRNA/PTEN mediated regulatory SASP exists in senescent CAF of OSCC In absence of cisplatin treatment SA-β-gal activity is minimal in CAF obtained from genetically stable tumours; CAF/GS-OSCC (BICR69 and BICR70) (n=2). CAF derived from genetically unstable tumours; CAF/GU-OSCC (BICR63 and BICR18) are senescent and are positive for SA-β-gal activity irrespective of passage number (n=3). Cisplatin induces SA-β-gal activity in non-senescent CAF/GS-OSCC and amplifies its activity in the senescent CAF/GU-OSCC, n=3 (**A**). Senescent CAF/GU-OSCC (n=3) secreted more MCP-1 (**B**) and IL-6 (**C**) than non-senescent CAF/GS-OSCC (n=2) and normal (n=4) control fibroblasts. Senescent CAF/GU-OSCC stimulated migration of both D20 (**D**) and H357 cells (**E**) *in vitro* (n=3). Invasion of H357 cells was also increased in presence of senescent-CAF in organotypic models (BICR63, n=3) (**F**); immunohistochemistry for p16^INK4A^ indicated senescence in fibroblasts incorporated in the organotypic model. qRT-PCR showed miR-335 (**G**) levels are elevated in CAF of both GS-OSCC (n=2) and GU-OSCC (n=4) tumours. Western blot showed PTEN expression was reduced in senescent CAF/GU-OSCC (n=4) compared to non-senescent CAF/GS-OSCC (n=2) and normal fibroblasts (n=6) (**H-I**). All experiments were performed independently as indicated by n and with technical repeats. The bars represent mean ± SEM or mean ± STDEV (**G**). *p<0.05, by one-way ANOVA with post-hoc correction by Holm-Sidak method (**C**, **G**, **I**) and Dunn's method **(B)**, two-way ANOVA with post-hoc corrections by Bonferroni t-test (**D-E**). (See supplementary Figure S6-S7).

We have previously reported that CAF isolated from aggressive, genetically unstable, squamous cell carcinomas have a predominantly senescent phenotype [4]. We next utilized this knowledge to examine whether a COX-2/miR-335/PTEN signaling cascade contributes to the development of the SASP associated with senescent CAF as well as normal fibroblasts induced to senesce. Even though these CAF does not bear any genetic abnormalities and are diploid in nature [36] initially we investigated that the non-senescent CAF of genetically stable tumours (CAF/GS-OSCC) did not by-pass senescence by treating them with cisplatin. Treatment of senescent CAF from genetically unstable tumours (CAF/GU-OSCC) with cisplatin further reinforced SA β-galactosidase activity and induced senescence in non-senescent CAF from genetically stable tumours (CAF/GS-OSCC) (Fig 6A, Fig S6). Analysis of conditioned media collected from these cells revealed the senescent CAF/GU-OSCC released higher levels of MCP-1 and IL-6 than both non-senescent CAF/GS-OSCC and normal proliferating fibroblasts (Fig 6B-C). The senescent CAF/GU-OSCC were also able to induce more dysplastic and malignant cell migration and invasion than normal fibroblasts and non-senescent CAF/GS-OSCC, and this was augmented by cisplatin treatment (Fig 6D-F). Examination of SASP-associated miRNA by qRT-PCR revealed levels of miR-335 was significantly elevated in senescent CAF/GU-OSCC compared to normal proliferating fibroblasts (n=4). Non-senescent CAF/GS-OSCC also demonstrated a significant but highly variable elevation of miR-335 compared to normal fibroblasts (Fig 6G). Analysis of PTEN protein expression in CAF by western blot showed the senescent CAF/GU-OSCC expressed significantly less PTEN protein compared to the non-senescent CAF/GS-OSCC and normal fibroblasts (Fig 6H-I).

A similar reduction in PTEN expression was observed in senescent CAF isolated from colorectal cancer patients compared to normal fibroblasts isolated from the adjacent non-cancerous bowel section of the same individuals underscoring its importance in stromal evolution in colorectal cancer (n=15, Fig S7A-B).

**Figure 7:**
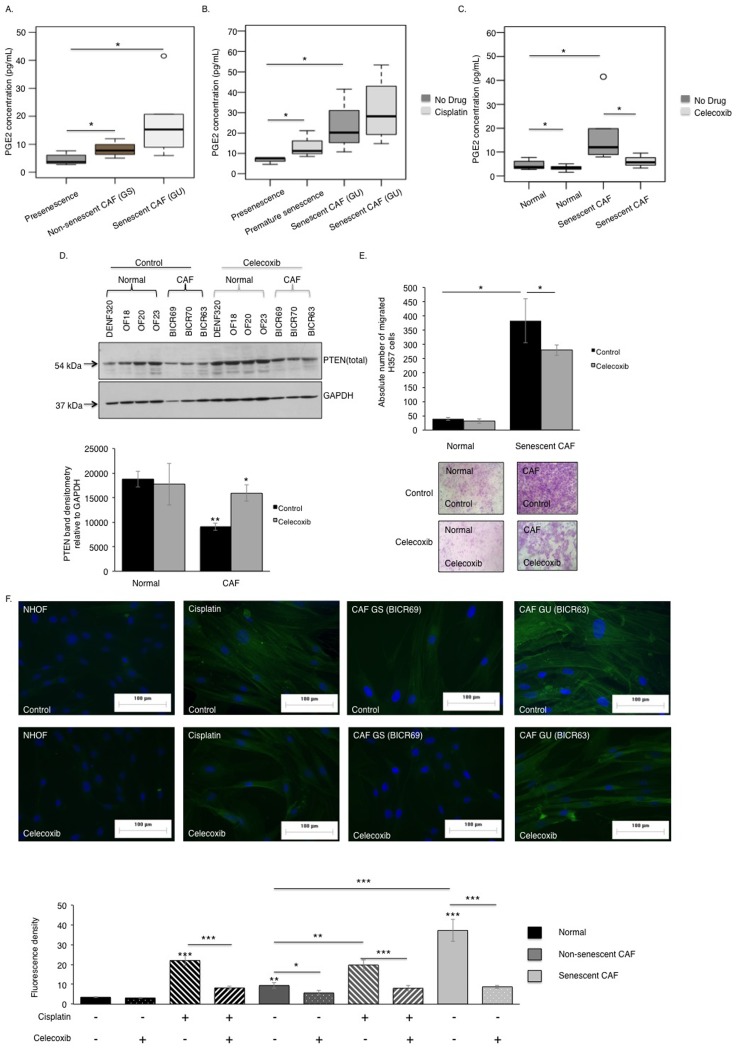
The COX-2/PTEN axis regulates the pro-tumourigenic phenotype of senescent CAF Senescent CAF/GU-OSCC (n=3) secreted more PGE2 than the non-senescent CAF/GS-OSCC (n=2) and normal fibroblasts (n=3), as assessed by ELISA (**A**). Cisplatin treatment increased PGE2 secretion by senescent CAF and normal fibroblasts (n=3) (**B**). Blockade of COX-2 catalytic activity diminished PGE2 secretion by normal fibroblasts and senescent CAF (n=3) (**C**). In contrast to normal fibroblasts (n=4), senescent-CAF (n=3) expressed less PTEN protein and this was rescued by celecoxib treatment (**D**). Celecoxib treated senescent CAF showed reduced capacity to stimulate paracrine migration of H357 cells *in vitro,* (n=3) (**E**). Celecoxib treatment abrogated activation of CAF and cisplatin-induced premature senescent fibroblasts by extenuating stress fibers formation visible as diminished fluorescence intensity (n=3) (**F**). All experiments were performed independently as indicated by n and with technical repeats. The bars represent mean ± SEM. *p<0.05, by Mann-Whitney U-test (**A**), two-way ANOVA with post-hoc correction by Bonferroni t-test (**B**-**C**) and Holm-Sidak method (**E**) and paired student's t-test (**D**).

Although miR-335 levels demonstrated a trend towards an increase this failed to reach statistical significance in the cohort utilized (p=0.494) (Fig S7C).

Determination of COX-2 activity in senescent CAF revealed COX-2 expression and PGE2 secretion was amplified in these cells compared to non-senescent-CAF and normal fibroblasts (Fig 7A). PGE2 levels were further increased by treating these cells with cisplatin (Fig 7B), and this increase was inhibited by celecoxib (Fig 7C). Celecoxib treatment restored PTEN levels in senescent CAF/GU-OSCC to approaching the levels in proliferating normal fibroblasts (Fig 7D). Chemotaxis of cancer cells towards conditioned media derived from celecoxib treated senescent CAF was reduced and the intensity of fluorescence signal from α-SMA positive stress fibers became attenuated and less conspicuous in these fibroblasts (Fig 7E-F).

## DISCUSSION

The tumour microenvironment is increasingly recognized as a potential target for novel therapeutics. This is hampered, however, by the lack of understanding of the mechanisms leading to the corruption of the predominant cell type in the tumour stroma, the fibroblast. Here we demonstrate widespread alterations in miRNA expression occur in fibroblasts induced to senesce by cisplatin, a widely used chemotherapeutic agent. These changes in miRNA expression coincide chronologically with the development of a pro-tumourigenic secretory phenotype (the senescence-associated secretory phenotype, SASP). We show that a miRNA elevated in concert with the development of the SASP, miR-335, contributes to the generation of a pro-inflammatory SASP by increasing the release of MCP-1, IL-6, and MMP-2, at least in part by down-regulating PTEN. The aberrant expression of miR-335 and PTEN is restored by treatment with celecoxib, a specific inhibitor of COX-2, indicating a role for prostaglandin signaling in the generation of the SASP in response to cisplatin. miR-335 was found to be up-regulated in CAF isolated from oral cancers, and this was associated with increased PGE2 secretion and reduced PTEN expression.

The majority of reports of roles for microRNA in CAF to date have focused on the development of contractile, α-smooth muscle expressing myofibroblastic CAF [e.g. 37]. Fewer studies have examined the contribution of microRNA to the secretory phenotype observed in senescent CAF. Bhaumik et al. identified up-regulation of miR-146a in senescent fibroblasts [18], and proposed its involvement in suppression of IL-6 by targeting IRAK1. Here we also observed up-regulation of miR-146a but also identified aberrant expression of a number of other microRNA, including miR-335, associated with SASP development in primary human fibroblasts. *In silico* analysis indicated that PTEN was a putative target of miR-335, which was subsequently confirmed by reporter assay and analysis of PTEN protein levels in fibroblasts over-expressing miR-335. PTEN is a negative regulator of PI3 kinase/Akt signaling, originally identified as a tumour suppressor in epithelial cells [38]. Its function in mesenchymal cells is less well understood but it is reported to be a key regulator of myofibroblast differentiation [39]. Furthermore, PTEN is reported to be down-regulated in the stroma of breast and oropharyngeal tumours [40, 41] and to play a role in regulating stromal fibroblast-epithelial interactions, but the underlying mechanisms are unclear [27]. Although previously implicated in the development of senescence, this is the first study to identify a role for PTEN in regulating the SASP of senescent fibroblasts. The ability of PTEN-depleted fibroblasts to enhance tumour cell migration and its down-regulation in senescent CAF (derived from both oral and colorectal tumours) suggest that PTEN may be a critical modulator of pro-tumourigenic signaling in the tumour microenvironment and contribute to chemotherapy resistance and cancer recurrence or progression in patients receiving chemotherapy and radiotherapy. The mechanisms by which PTEN regulates the SASP remain obscure; in our system transient depletion of PTEN by siRNA resulted in a significant increase in the expression of MMP2 and a trend towards increasing secretion of IL-6 but not any other SASP factors. We suggest this may have been the result of the transient nature of the knockdown of PTEN; we are currently utilizing CRISPR to generate pten null fibroblasts and expression vectors to stably over-express PTEN in oral fibroblasts in order to examine further the role of PTEN in the generation of the SASP.

miR-335 was first described as a tumour suppressor and has since been ascribed roles in diverse pathologies such as depression and osteoarthritis [42, 43]. Notably, miR-335 has been implicated in the generation of senescence in mesangial cells [20] and mesenchymal stem cells [22]; in both of these studies, miR-335 was found to increase upon provocation of senescence, in keeping with our findings in primary fibroblasts. In addition, Tomé et al observed IL-6 and MCP-1 secretion was increased in MSC over-expressing miR-335 [22], further validating the suggestion that miR-335 may play a key role in SASP generation. In our study, we observed no effect of miR-335 overexpression on the acquisition of markers of senescence, in contrast to the findings of Tomé et al, possibly reflecting differences between species and cell types in the functions of miR-335. In our study, miR-335 was expressed at higher levels on the provocation of senescence, and this increased with time, following a similar time course to the increase in secretion of SASP proteins. This, together with the observation that over-expression of miR-335 increases the release of SASP factors, suggests miR-335 may be a novel regulator of the SASP. This hypothesis is further supported by elevated expression of miR-335 in senescent CAF, which also display an enhanced SASP. Surprisingly, miR-335 levels were also elevated, to a variable degree, in non-senescent CAF. We speculate this may reflect the inherent heterogeneity in the CAF phenotype compared to normal fibroblasts.

We have previously shown that COX-2 expression increases aberrantly in cancer cells in response to stromal cues [44]. Although elevated prostaglandin generation by senescent cells has been reported [30], here we demonstrate that a selective COX-2 inhibitor can suppress the pro-inflammatory SASP of senescent CAF and normal fibroblasts, and to link COX-2 activity with PTEN expression levels in senescent fibroblasts. Taken together these data suggest the existence of a novel mechanism, which in part appears to involve post-transcriptional regulation of PTEN by miR-335, that could be susceptible to stromally-directed therapeutic intervention to ameliorate the off-target/bystander detrimental effects of chemotherapy agents.

## METHODS

### Cell culture

Normal human primary oral fibroblasts were extracted from gingiva of patients attending Charles Clifford Dental Hospital for tooth extraction (approved by Sheffield Research Ethics Committee; 07/H1309/105), via collagen digestion and selective trypsinization. Normal oral fibroblasts, oral squamous cell carcinoma (OSCC) derived cell lines SCC4 and H357 and oral dysplasia derived cell line D20 (kindly provided by Dr K. Hunter, University of Sheffield, UK) and cancer-associated fibroblasts (CAF) derived from OSCC [36] were cultured in DMEM supplemented with 10% (v/v) fetal calf serum (FCS) and 2 mM L-glutamine. All cultures were incubated at 37oC and 5% CO2. Normal dividing cells (presenescent or proliferating) had a cell cycle time of 48-54 hours and senescent cells, including senescent CAF, >14 days. CAF and control fibroblast from macroscopically normal tissue from colorectal cancer patients were kindly gifted by Professor John McCall from the Department of Surgical Science of Dunedin Hospital, New Zealand. This study had been approved by the Human Ethics Committee of the University of Otago (14/NTA/33).

### Induction of senescence

Early passage fibroblasts (10 mean population doublings [8]) were induced to senesce prematurely with sub-cytotoxic doses of H_2_O_2_ (500 μM) for 2 h and cisplatin (10 μM) for 24 h. After completing the duration of treatments the fibroblasts were washed twice with 1X PBS (Sigma) and fresh normal growth media were added. The cells were then cultured for a time-point of 15 days with regular changes of media every 72 h in both the control and drug-induced fibroblasts. In parallel oral fibroblasts from the same patients were cultured and sub-cultured continuously until a mean population doublings of 80 (replicative exhaustion) to yield replicative senescent cells [8] (>85% cells are positive for senescence associated β-galactosidase activity). Acquisition of senescence was examined by analyzing senescence associated β-galactosidase (SA β-Gal) activity at day 15 post-treatment with genotoxic stimuli and on replicative senescent oral fibroblasts. In addition, qPCR and immunocytochemistry were used to analyze the levels of cell cycle markers p16^INK4A^ and p21^CIP1^, respectively, as described below.

### Transient transfection of fibroblasts

Fibroblasts (5 × 10^5^) were seeded in T25 flasks and incubated overnight. These cells were either transfected with non-targeting miRNA negative control and specific synthetic miRNA precursors (Life Technologies) or with PTEN siRNA and silencer Cy3 labelled negative control 1 siRNA (Life technologies) at a final con-centration of 50 nM using Oligofectamine 2000 (Life Technologies) in reduced serum media. Total RNA and protein were extracted at 72 h post-transfection.

### Luciferase reporter assay

A 1345 bp fragment of the PTEN 3′UTR containing the putative binding site for miR-335 was amplified from cDNA extracted from primary oral fibroblasts using the following primers: forward 5′ ACTGAACTAGTTGTTGACACGTTTTCCATACCTT 3′ and reverse 5′ TTATTGAGCTCGGGATGAGGCATTATCCTGTACAC 3′. Amplicons were digested with Sac I and Spe I and ligated into pmiR-Report (Life Technologies), immediately downstream of the firefly luciferase coding region under the control of the CMV promoter. The recombinant plasmid, pmiR-PTEN (1 μg) was co-transfected with pSV-β-galactosidase control vector (0.6 μg) along with control non-targeting miRNA or miR-335 precursor (50 nM) using Fugene HD (Promega), according to the manufacturer's instructions. After 48 h incubation, cells were washed with PBS and lysed using reporter lysis buffer (Promega). Luciferase activity was measured using the Stop and Glo assay (Promega), according to the manufacturer's instructions. This was normalized to the β-galactosidase activity of the same sample using the β-galactosidase Enzyme Assay System (Promega) according to the manufacturer's protocol.

### Preparation of conditioned medium

Fibroblasts were washed twice with PBS and incubated in serum-free media at 37^0^ C for 24 h. In cisplatin-treated and miRNA/siRNA transfected oral fibroblasts conditioned medium (CM) was prepared at day 15 post-treatment and 24 h post-transfection, respectively. Prior to performing every functional assay and measuring the levels of secreted cytokines and MMP-2 activity the CM of the senescent and presenescent fibroblasts were normalized to the cell density of 5 × 10^5^ per ml of CM. The synthetic oligonucleotide mimics had no effect on fibroblast proliferation and CM normalization to biomass (cell number) was not required.

### Tiling low-density arrays

Total RNA was extracted using mirVana miRNA isolation kit (Life Technologies). miRNA expression profiling was performed using commercially available Tiling Low-Density Array; TLDA (Life Technologies) which comprises of hybridization of pre-amplified cDNA against 754 human miRNA assays labeled onto microfluidic cards by TaqMan qRT-PCR. The raw data were extracted using RQ manager version 1.2.1 and analyzed by Data Assist Software version 3.0 (Life Technology). The ΔC_t_ values were normalized to U6 endogenous control at a C_t_ cut-off value of 34. Fold change of the differentially expressed miRNAs were calculated from ΔΔC_t_ values relative to the presenescence control.

### miRNA and gene expression assays

Following cDNA synthesis TaqMan and SYBR-Green probes and primers (Life Technologies) were used to validate candidate miRNAs and gene expression changes respectively in presenescent and prematurely senescent oral fibroblasts by qRT-PCR. The ΔC_t_ values were calculated by normalizing to U6 and RNU48 endogenous controls. The ΔΔC_t_ values of target genes were calculated by normalizing their levels to presenescence control. The mRNA expression is represented graphically as the mean fold change (2^−ΔΔC^_t_).

### Human cytokine antibody array

The soluble cytokines of SASP from cisplatin-treated and presenescent oral fibroblasts were determined by human cytokine antibody array 6 (Raybiotech). The signal intensities were quantified using 1D gel analysis software (BioRad).

### MCP-1, IL-6, and PGE2 ELISA

Human MCP-1 and IL-6 ELISA development kit (Peprotech) were used to analyze the levels of secreted MCP-1 and IL-6 in CM obtained from presenescent and cisplatin treated oral fibroblasts according to manufac-turer's protocol. Secreted levels of human PGE2 were determined using competitive ELISA (R&D system; KGE004B) according to manufacturer's instruction.

### Proliferation assay

SCC4, H357 and D20 cells were seeded down at 5 × 10^3^ per 100 μl of DMEM into each well of a 96-well plate. The cells were serum starved overnight and subsequently treated with freshly prepared CM of senescent and presenescent oral fibroblasts. The rate of proliferation was measured at time points 0 - 120 h, using CyQuant NF cell proliferation assay kit (Invitrogen, #C35006) according to the guidance of the manufacturer. Fluorescence was recorded at an excitation of 485 nM and emission detection at 530 nm using a microplate reader (Tecan).

### Migration and invasion assays

SCC4, H357 and D20 cells were serum starved overnight prior to experimentation. Cells were trypsinized and resuspended in DMEM containing 0.1% (w/v) BSA at 5 × 10^5^ cells/ml. Cell suspension (200 μL) was pipetted into the top of the transwell chamber with/without matrigel coating and CM was added to the lower chamber. For blockade of secreted MCP-1, CM was incubated with either anti-MCP-1 antibody (20 μγ/ml, subtype IgG, R&D: MAB679) or isotype IgG1 and IgG2 controls (Serotec: MCA1209, Invitrogen: MG2b00) 1 h preceding chemotaxis assay. SCC4 and D20 cells were allowed to migrate for 20 h and H357 cells for 40 h. The cells were then swabbed away from the inside of the transwell chamber and the cells adhering to the underside of the chamber were fixed with 100% (v/v) methanol. Migrated cancer and dysplastic cells were stained with 0.1% (w/v) crystal violet. The cells were counted by light microscopy at 40x magnification. Three fields of view from each insert were counted. Invasion was measured from invasive index using the formula:

$$I_{i}=\left|\left(T_{m(t)}{-T}_{i(t)}\right)\right|/\left|\left(T_{m(c)}{-T}_{i(c)}\right)\right|$$

where I_i_ stands for invasive index, T_i_ is the total number of invading cells, T_m_ is the total number of migrated cells, *t* is treatment with different types of CM and *c* is control.

A modified 3D organotypic model [16] was used to examine the invasive potential of H357 cell lines into the human de-epithelialized dermis in the presence of normal, cisplatin-induced and CAF (manuscript under preparation). The model was grown in an air-liquid interface for 14 days. These were then formalin (10%) fixed, paraffin embedded and microdissected. Haematoxylin and eosin staining of tissue sections were used to assess the depth of invasion of cancer cells.

### Western blot analysis

Oral fibroblasts were lysed in RIPA lysis buffer (Sigma) supplemented with protease and phosphatase inhibitor cocktail (Roche) and benzonuclease (Sigma). Protein was quantified by Pierce BCA protein assay kit (Thermoscientific). Membranes were incubated with human rabbit monoclonal antibody for total PTEN (#9559), pan-AKT (#4691), phospho-AKT (ser473) (#4060) and phospho-AKT (thr308) (#2965) and GAPDH (#G9545) (Cell Signaling) at 1 in 1000 and human mouse monoclonal antibody β-actin (Sigma) at 1 in 10000. Mouse and rabbit IgG conjugated with horseradish peroxidase (Sigma, 1 in 3000) were used as secondary antibody. Chemiluminescence was recorded on X-ray films using an automated X-ray film processor (Xograph imaging systems). Band densitometry was analyzed using 1D gel analysis software (BioRad).

### Gelatin zymography

Fresh conditioned media was collected from fibroblasts and concentrated using vivaspin-500 5 kDa cut-off spin columns (Sartorius, #VS0101) by centrifugation at 10,000 rpm for 15 m. Concentrated conditioned media from senescent and presenescent fibroblasts were mixed with 2X non-reducing zymography sample loading buffer (Tris-HCl, 200 mM; SDS, 2% (w/v); glycerol, 20% (v/v) and bromophenol blue 0.1% (w/v), pH 6.8), and incubated for 30 min at 37oC. Samples were electrophoresed in polyacrylamide gels containing gelatin (10% (w/v)). Following electrophoresis gels were allowed to renature in 2.5% (v/v) Triton X-100 in PBS for 1 h and maintained in zymogram developing buffer; 0.5 M Tris, 2 M NaCl (BDH GPR), 50 mM CaCl_2_ (Sigma), 50 μM ZnCl_2_ (Sigma), 1% (v/v) Triton X-100, pH=7.5; at 37°C for 24 h with gentle agitation. Gels were then stained with Coomasie brilliant blue R-250 for 30 m and destained with 40% (v/v) methanol and 10% (v/v) glacial acetic acid until distinct faint bands became visible. Fisher Scientific's EZ-Run pre- stained Rec protein ladder was used as molecular weight markers. Gelatinase activity was determined using Quantity One 1D gel software (BioRad; version 4.5.0) and the intensity of bands were divided by the total number of fibroblasts present in each sample of conditioned medium to normalize to cell density.

### Immunocytochemistry and histological analysis

Paraffin-embedded tissues were deparaffinised. Microwave heat mediated antigen retrieval in citrate buffer was required for p16^INK4A^ (JC8, Santa Crutz, 1 in 100 in 0.5% normal horse serum) antibody. The tissue sections were counterstained using haematoxylin. Fibroblast activation was determined by direct immuno-fluorescence cytochemistry for α-SMA using FITC conjugated anti-mouse monoclonal α-SMA antibody (Sigma, 1 in 100 dilutions in 5% bovine serum albumin). The fixed cells were mounted in DAPI rich Prolong Gold anti-fade reagent and fluorescence was observed in Zeiss microscope (Axioplan 2 imaging) and photographed using image pro-plus software version 7.0.1, at 40X magnification. Fluorescence intensity was quantified using NHS Image J software (version 1.49).

### Inhibition of COX-2 activity

Senescent and non-senescent oral fibroblasts were treated with the selective COX-2 inhibitor celecoxib (1 μM) for 48 h in serum free DMEM. Untreated cells were used as the control.

### Bioinformatics database mining

Target Scan version 6.2 (http://www.targetscan.org) and miRwalk (www.umm.uni-heidelberg.de/apps/zmf/mirwalk/) were used to predict putative gene targets of candidate miRNA of interest. DIANA-miRPath online tool version 2.0 was used to predict the miRNA-targeted pathways that are altered in senescent fibroblasts.

### Statistical analysis

Sigma Plot (version 12) was used to determine statistical significance. One-way ANOVA, Two-way ANOVA, ANOVA with repeated measures and paired student's t-test were used to analyze data. Appropriate posthoc corrections were used according to data distribution and experimental procedures as described in figure legends. Mann-Whitney U-test was performed when data were not normally distributed. P-value <0.05 was considered as statistically significant wherein *p<0.05, **p<0.01 and ***p<0.001.

## SUPPLEMENTAL DATA FIGURES AND TABLES


