# Supplementary material for: A miR-335/COX-2/PTEN axis regulates the secretory phenotype of senescent cancer-associated fibroblasts
Source: Aging (Albany NY). 2016 Jun 29;8(8):1608–24. doi: 10.18632/aging.100987 (PMC5032686; doi:10.18632/aging.100987)
Supplement: Supplementary file 1 [file aging-08-1608-s001.pdf]

## SUPPLEMENTARY DATA

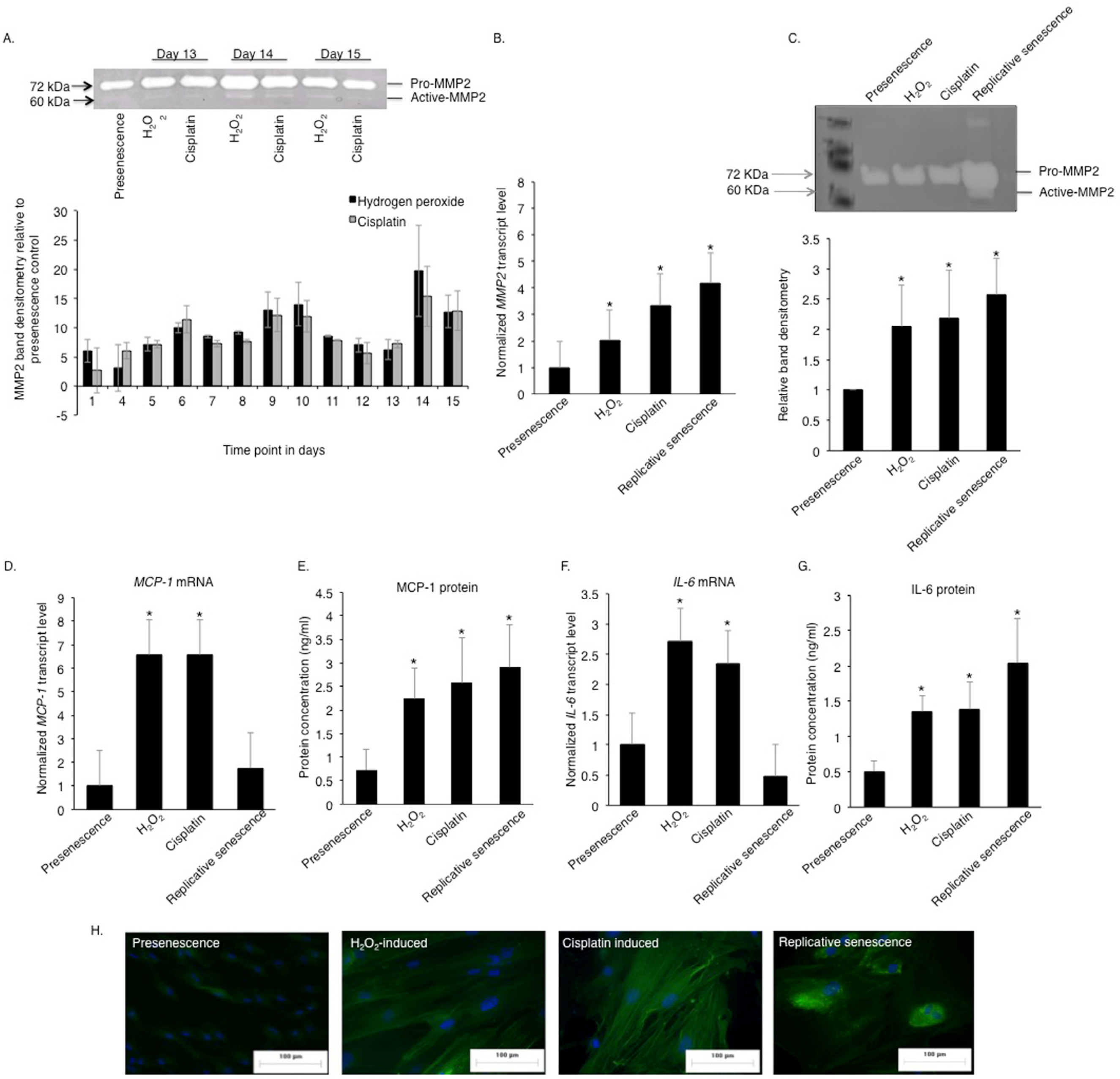


**Figure S1. Validation of the pro-tumourigenic SASP of senescent oral fibroblasts.** Gelatin zymography using conditioned media of oral fibroblasts post-induction of senescence demonstrated a gradual increase in the amounts of MMP-2 in senescent fibroblasts during acquisition of senescence and establishment of SASP (**A**). The conditioned media were normalized to 5 X $10^5$ cells/ml. In addition, qRT-PCR (**B**) and gelatin zymography (**C**) in fibroblasts induced to senesce using different stimuli corroborated with initial findings and further showed senescent oral fibroblasts synthesized and secreted more active MMP-2 than presenescent proliferating controls (n=3). Prematurely senescent oral fibroblasts also expressed and secreted more MCP-1 (**D**-**E**) and IL-6 (**F**-**G**) than proliferating controls, confirmed by qRT-PCR and ELISA. Despite of having lower MCP-1 and IL-6 mRNA levels, the replicative senescent fibroblasts secreted more of these proteins than proliferating control after normalizing the secreted protein to cell number (n=3) (**D-G**). Direct immunofluorescent cytochemistry showed senescent oral fibroblasts reorganizes and expressed more $\alpha$-SMA positive actin filaments (n=3) (**H**). All experiments were performed independently as indicated by n and with technical repeats. The data represents mean ± STDEV (**A**,**B**,**D**,**F**) or mean ± SEM (**C**,**E**,**G**) of three independent experiments in triplicate. *p<0.05, by one-way ANOVA with post-hoc corrections by Dunn's method (**B**-**G**).

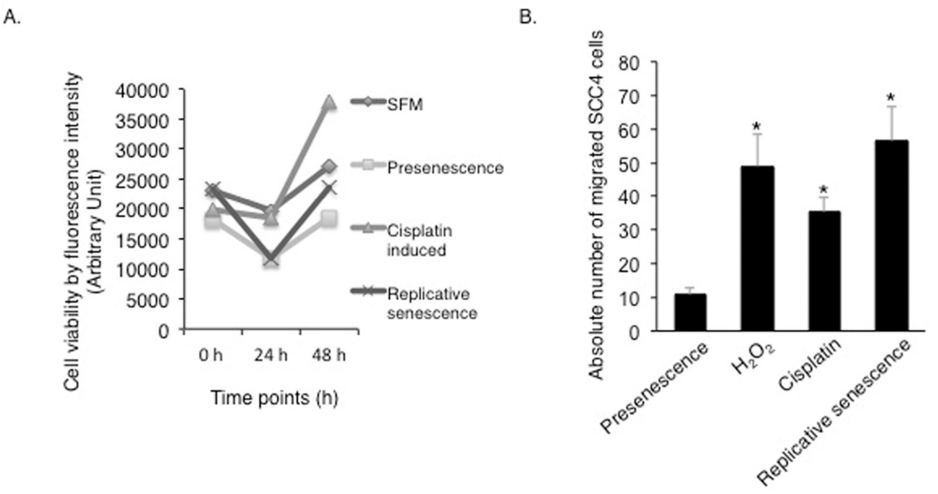


**Figure S2. The SASP mediated paracrine cross-talk between senescent oral fibroblasts and cancer cells does not demonstrate cell line specificity.** Soluble factors secreted into conditioned media of senescent fibroblasts stimulated proliferation (**A**) and migration (**B**) of another oral squamous cell carcinoma derived cell line SCC4 *in vitro* (n=3). All experiments were performed independently as indicated by n and with technical repeats. The data represents mean ± SEM of three independent experiments in triplicate. *p<0.05, for proliferation assay the data were analyzed by two-way repetitive measure ANOVA with post-hoc corrections by Holm-Sidak method (**A**) and for migration assay the data were analyzed by one-way ANOVA with post-hoc corrections by Dunn's method (**B**).

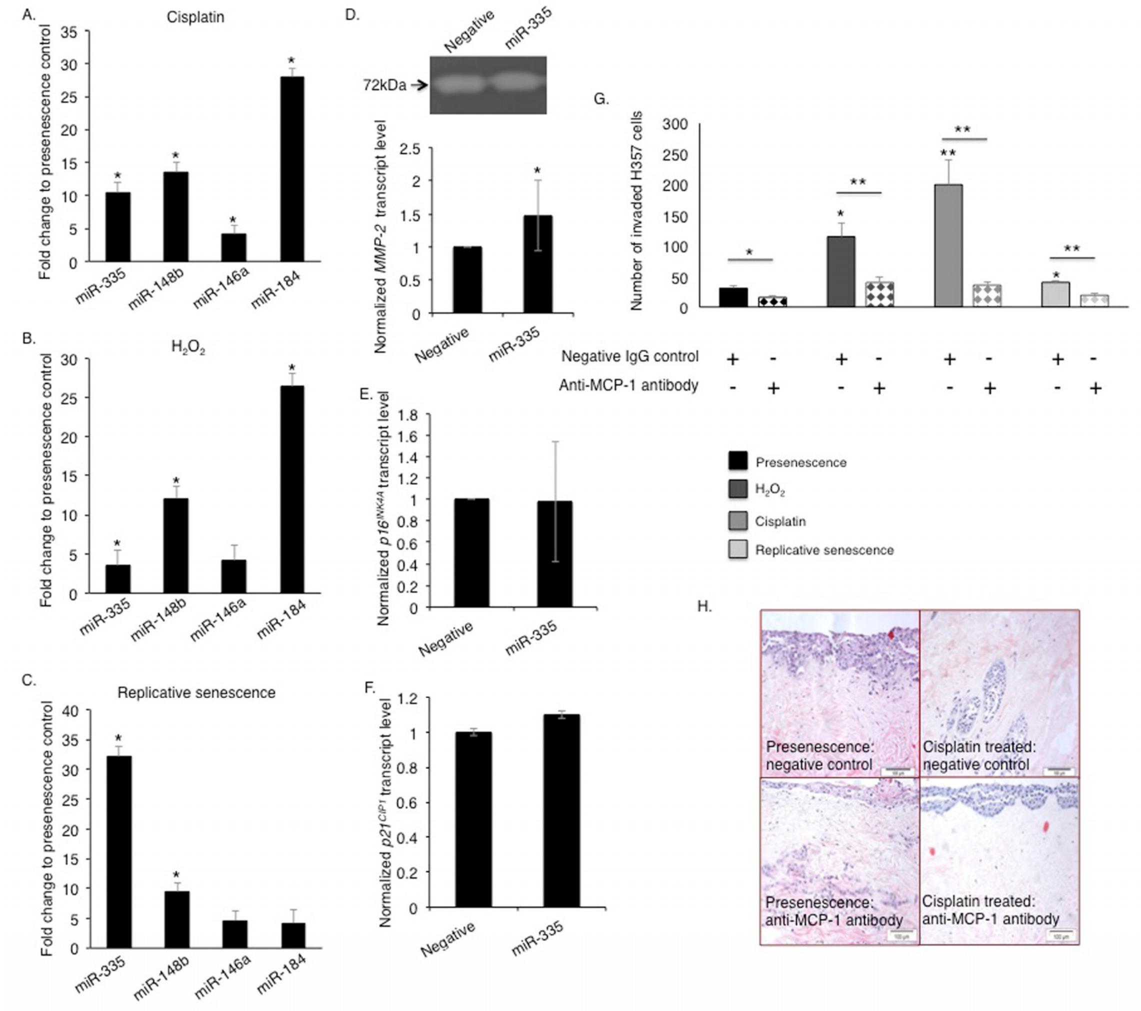


**Figure S3. The pro-tumourigenic SASP of senescent fibroblasts is associated with differential expression of miRNAs.** qRT-PCR was used to validate expression levels of miR-335, miR-148b, miR-146a and miR-184 in cisplatin induced senescent fibroblasts (**A**), $H_2O_2$ induced senescent fibroblasts (**B**) and replicative senescent oral fibroblasts (**C**) compared to proliferating control (n=3). miR-335 transfected oral fibroblasts expressed more MMP-2 (**D**). Examinations for markers of cell cycle arrest showed miR-335 transfected oral fibroblasts (n=3) did not display any alterations in levels of $p16^{INK4a}$ (**E**) and $p21^{CIP1}$ (**F**). Blockade of secreted MCP-1 in conditioned media of senescent oral fibroblasts significantly impeded invasion of H357 cells *in vitro* (n=3) (**G**). All experiments were performed independently as indicated by n and with technical repeats. The data represents mean ± STDEV (**A**-**D**) or mean ± SEM of three independent experiments in triplicate. *p<0.05, by one-way ANOVA with post-hoc corrections by Holm-Sidak method (**A**-**C**), paired student's t-test (**D**-**G**).

A.

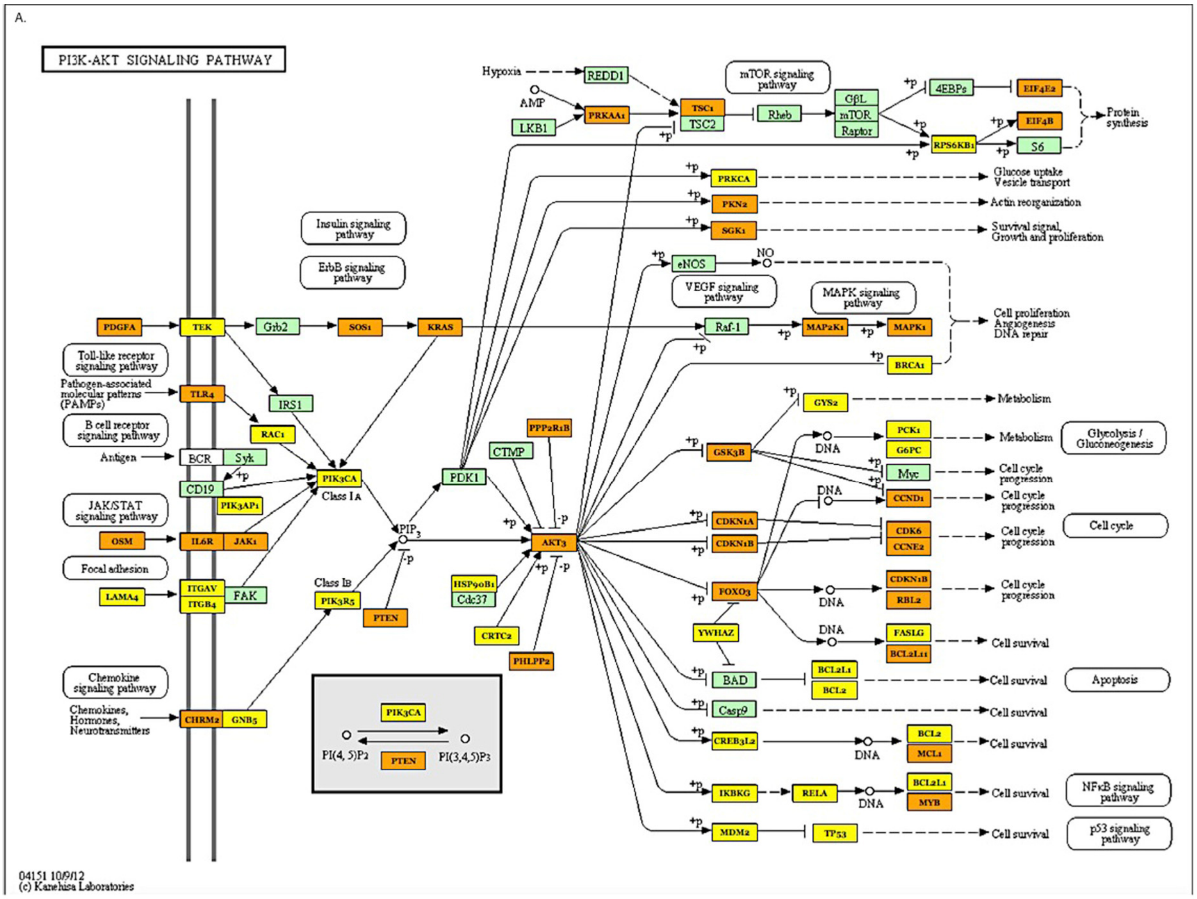


B.

**List of microRNAs interacting in PI3K-Akt signaling pathway (hsa04151)**

| miRNA | #Genes |
|---|---|
| hsa-miR-335 | 1 |
| hsa-miR-34c-59 | 8 |
| hsa-miR-146a-5p | 3 |
| hsa—miR-625-5p | 13 |
| hsa-miR-137 | 22 |
| hsa-miR-301b | 11 |
| hsa—miR-519a-3p | 21 |
| hsa-miR-222-3p | 6 |
| hsa-miR-512-3p | 7 |
| hsa-miR-508-3p | 1 |
| hsa-miR-34a-5p | 17 |
| hsa-miR-409-5p | 2 |
| hsa-miR-219-1-3p | 3 |
| hsa-miR-7d-5p | 11 |
| hsa-miR-597 | 12 |
| hsa-miR-548d-3p | 52 |
| hsa-miR-616-3p | 5 |
| hsa-miR-216b | 18 |
| hsa-miR-28-5p | 1 |
| hsa-miR-885-5p | 4 |
| hsa-miR-199b-5p | 4 |
| hsa-miR-185-5p | 13 |
| hsa-miR-504 | 4 |
| hsa-miR-20a-5p | 25 |
| hsa-miR-29a-3p | 33 |
| hsa-miR-489 | 1 |
| hsa-miR-127-5p | 3 |
| hsa-miR-217 | 13 |

C.

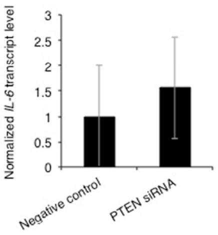


**Figure S4. miRNA pathway analysis showing the gene targets of miRNAs interacting with PI3 Kinase/Akt pathway in senescent fibroblasts and the effect of transient knockdown of PTEN on IL-6 expression in oral fibroblasts.** DIANA miR-Path analysis tool was used to predict and identify the gene targets of differentially expressed miRNAs affecting the PI3 kinase/Akt pathway in senescent fibroblasts wherein yellow highlights indicate genes targeted by one miRNA, orange indicates genes targeted by two miRNAs and red indicates genes targeted by three or more miRNAs (**A**-**B**)**.** qRT-PCR analysis of cDNA synthesized from fibroblasts having transient knockdown of PTEN demonstrated no significant difference in IL-6 mRNA levels in comparison to control fibroblasts transfected with non-targeting siRNA, n=3 (**C**). All experiments were performed independently as indicated by n and with technical repeats.

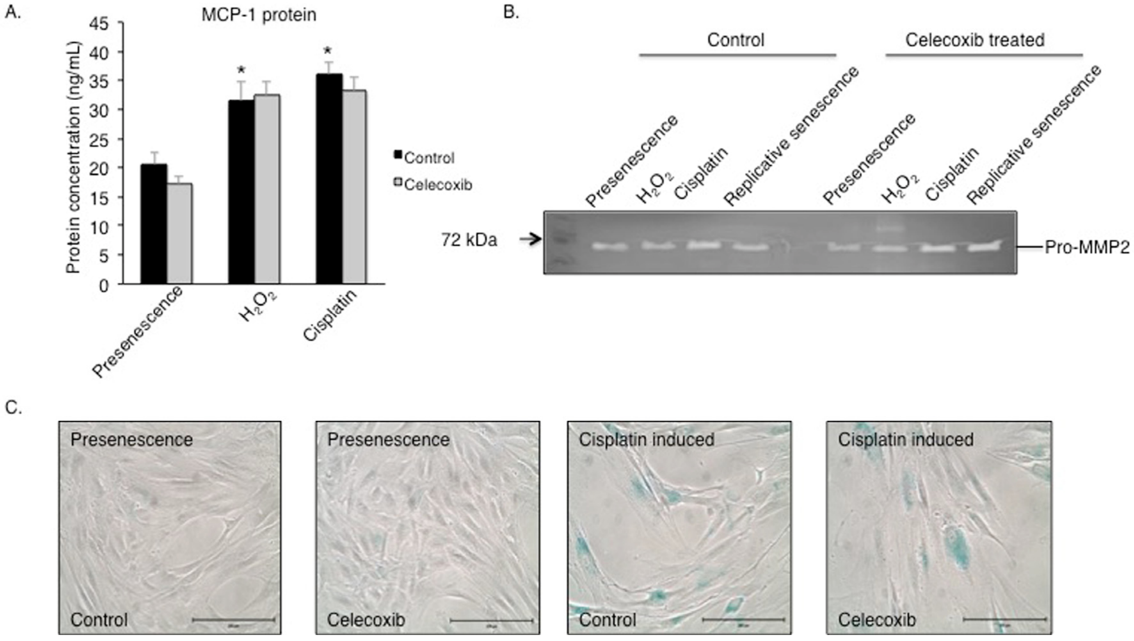


**Figure S5. MCP-1 and MMP-2 secretion by senescent fibroblasts are independent of COX-2 activation.** Selective inhibition of COX-2 activity with celecoxib (1μM) in senescent fibroblasts failed to reduce secretion of MCP-1 (**A**) and MMP-2 (**B**) into the conditioned media (n=3). Celecoxib did not stimulate senescence in proliferating control fibroblasts and had no effect on the established senescent phenotype of cisplatin induced senescent fibroblast as determined by SA-β-gal activity (n=3) (**C**). All experiments were performed independently as indicated by n and with technical repeats. The data represents mean ± STDEV (**A**-**B**), *p<0.05, by paired student's t-test (**A**).

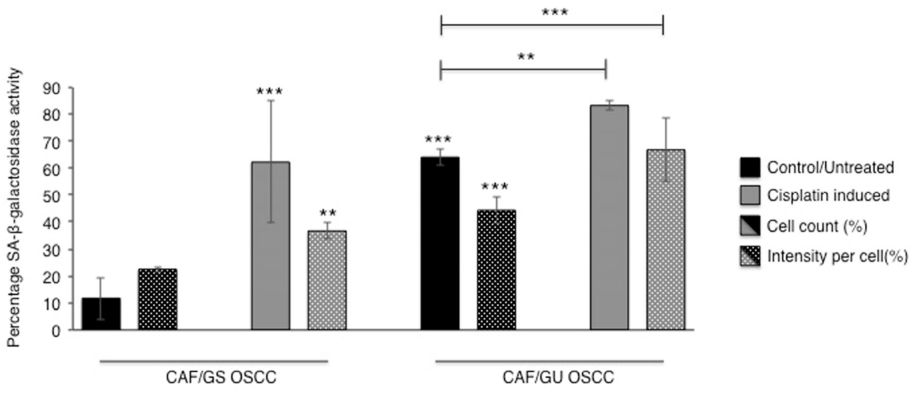


**Figure S6. Cisplatin induces senescence in non-senescent CAF/GS-OSCC and amplifies the senescent phenotype of senescent CAF/GU-OSCC.** SA-β-gal activity was assessed in CAF treated with cisplatin and untreated control. The number of SA-β-gal positive cells (blue) was counted and the intensity of blue colour per cell was also measured using Image J software (version 1.49) to determine senescence reinforcement. Paired student's t-test was used to determine statistical significance. *p<0.05 was considered statistically significant.

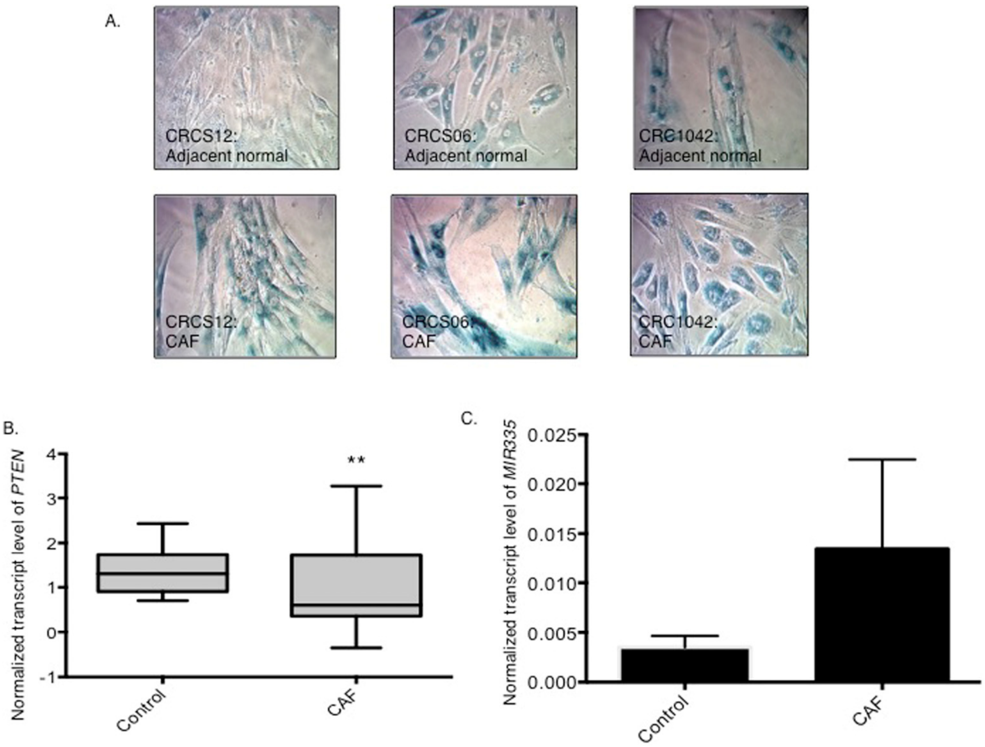


**Figure S7. The senescent CAF of CRC expresses less PTEN and more miR-335.** SA-β-galactosidase positive CAF was observed in CRC patients (n=15), a small population of control fibroblasts from the adjacent normal mucosa also demonstrated some positivity (**A**). qRT-PCR using cDNA synthesized from RNA isolated from CAF of colorectal cancer patients (n=15) showed a reduction in PTEN mRNA levels (**B**) and a trend towards increasing miR-335 transcripts levels ($p=0.556$). (**C**) The data represents mean ± STDEV of 15 patients in triplicate. *$p<0.05$ by paired student's t-test.

**Table S1. Differentially expressed miRNAs in cisplatin induced senescent fibroblasts compared to presenescent proliferating control.**

| miRNA ID | Fold change | P-value | Significance (No correction) | Significance (FDR=30%) | Holm-Sidak method correction |
|---|---|---|---|---|---|
| hsa-miR-223 | 705.1190 | 0.3724 | | | |
| hsa-miR-939 | 327.6360 | 0.3726 | | | |
| hsa-miR-1243 | 251.7600 | 0.3541 | | | |
| hsa-miR-663B | 147.8170 | 0.3512 | | | |
| hsa-miR-590-3P | 120.9350 | 0.3762 | | | |
| hsa-miR-452# | 109.6670 | 0.3729 | | | |
| hsa-miR-184 | 54.1033 | 0.2097 | | | |
| hsa-miR-1204 | 50.8617 | 0.3680 | | | |
| hsa-miR-888 | 37.2999 | 0.1824 | | | |
| hsa-miR-875-5p | 33.0642 | 0.3868 | | | |
| hsa-miR-1288 | 30.9889 | 0.2175 | | | |
| hsa-miR-363# | 26.9167 | 0.3697 | | | |
| hsa-miR-512-3p | 24.8025 | 0.0796 | | ** | |
| hsa-miR-1302 | 21.4167 | 0.3686 | | | |
| hsa-miR-517a | 15.3168 | 0.0011 | *** | *** | *** |
| hsa-miR-645 | 14.8657 | 0.4024 | | | |
| hsa-miR-361-3p | 14.0477 | 0.1279 | | | |
| hsa-miR-606 | 13.6667 | 0.3654 | | | |
| hsa-miR-662 | 13.5833 | 0.3739 | | | |
| hsa-miR-922 | 13.0833 | 0.3739 | | | |
| hsa-miR-520h | 12.1015 | 0.3824 | | | |
| hsa-miR-148b | 11.8641 | 0.3202 | | | |
| hsa-miR-943 | 11.8363 | 0.3397 | | | |
| hsa-miR-133a | 11.8127 | 0.3559 | | | |
| hsa-miR-519b-3p | 11.4114 | 0.2994 | | | |
| hsa-miR-885-5p | 11.2683 | 0.0798 | | | |
| hsa-miR-1292 | 11.0930 | 0.3842 | | | |
| hsa-miR-517c | 10.5354 | 0.0111 | * | *** | |
| hsa-miR-944 | 10.2789 | 0.3838 | | | |
| hsa-miR-1238 | 10.0833 | 0.3739 | | | |
| hsa-miR-586 | 9.7105 | 0.4175 | | | |
| hsa-miR-124# | 9.6545 | 0.3614 | | | |
| hsa-miR-200a | 9.3425 | 0.3882 | | | |
| hsa-miR-1296 | 9.1140 | 0.3598 | | | |
| hsa-miR-638 | 8.8922 | 0.3959 | | | |
| hsa-miR-141# | 8.2500 | 0.3589 | | | |
| hsa-miR-29b-2# | 8.2254 | 0.4046 | | | |
| hsa-miR-519a | 8.2154 | 0.0364 | * | ** | |
| hsa-miR-1228# | 8.2001 | 0.4093 | | | |
| hsa-miR-1206 | 8.0938 | 0.3854 | | | |

| | | | | | |
|---|---|---|---|---|---|
| **hsa-miR-1291** | 8.0446 | 0.3512 | | | |
| **hsa-miR-23a** | 7.8395 | 0.3994 | | | |
| **hsa-miR-206** | 7.6997 | 0.0993 | | | |
| **hsa-miR-891a** | 7.5516 | 0.2486 | | | |
| **hsa-miR-216b** | 6.7836 | 0.0636 | | | |
| **hsa-miR-202#** | 5.9830 | 0.4358 | | | |
| **hsa-miR-205** | 5.9156 | 0.1843 | | | |
| **hsa-miR-1262** | 5.8348 | 0.1545 | | | |
| **hsa-miR-548J** | 5.6334 | 0.4514 | | | |
| **hsa-miR-508-3p** | 5.4649 | 0.0682 | | ** | |
| **hsa-miR-608** | 5.3745 | 0.1733 | | | |
| **hsa-miR-892b** | 5.2233 | 0.2832 | | | |
| **hsa-miR-186#** | 4.7706 | 0.4258 | | | |
| **hsa-miR-489** | 4.4804 | 0.1189 | | | |
| **hsa-miR-503** | 4.4286 | 0.3429 | | | |
| **hsa-miR-520b** | 4.3208 | 0.3626 | | | |
| **hsa-miR-1208** | 4.2500 | 0.3401 | | | |
| **hsa-miR-363** | 4.1806 | 0.2905 | | | |
| **hsa-miR-1283** | 4.0391 | 0.4318 | | | |
| **hsa-miR-375** | 4.0066 | 0.3304 | | | |
| **hsa-miR-432#** | 3.9618 | 0.4897 | | | |
| **hsa-miR-337-3p** | 3.8538 | 0.4805 | | | |
| **hsa-miR-485-5p** | 3.8510 | 0.2738 | | | |
| **hsa-miR-1252** | 3.8056 | 0.3989 | | | |
| **hsa-miR-708#** | 3.6667 | 0.3325 | | | |
| **hsa-miR-668** | 3.6375 | 0.5055 | | | |
| **hsa-miR-664** | 3.5373 | 0.2048 | | | |
| **hsa-miR-380** | 3.5164 | 0.4186 | | | |
| **hsa-miR-449a** | 3.4240 | 0.2277 | | | |
| **hsa-miR-9#** | 3.4112 | 0.1756 | | | |
| **hsa-miR-522** | 3.2896 | 0.2398 | | | |
| **hsa-miR-302c** | 3.2708 | 0.3264 | | | |
| **hsa-miR-1245** | 3.2424 | 0.5132 | | | |
| **hsa-miR-377#** | 3.2379 | 0.3263 | | | |
| **hsa-miR-596** | 3.1857 | 0.4647 | | | |
| **hsa-miR-149#** | 3.1627 | 0.3412 | | | |
| **hsa-miR-92b#** | 3.0329 | 0.1659 | | | |
| **hsa-miR-1305** | 3.0206 | 0.5348 | | | |
| **hsa-miR-335** | 2.9835 | 0.0032 | ** | *** | |
| **hsa-miR-125b-2#** | 2.8790 | 0.3429 | | | |
| **hsa-miR-544** | 2.8605 | 0.1739 | | | |
| **hsa-miR-146a** | 2.8174 | 0.0031 | ** | *** | |
| **hsa-let-7c#** | 2.7549 | 0.2381 | | | |

| | | | | | |
|---|---|---|---|---|---|
| **hsa-miR-1269** | 2.7533 | 0.4819 | | | |
| **hsa-miR-33a** | 2.7118 | 0.4417 | | | |
| **hsa-miR-549** | 2.7037 | 0.4294 | | | |
| **hsa-miR-1290** | 2.7032 | 0.1722 | | | |
| **hsa-miR-938** | 2.6943 | 0.0355 | * | | |
| **hsa-miR-620** | 2.6696 | 0.0443 | * | | |
| **hsa-miR-302a#** | 2.6580 | 0.4943 | | | |
| **hsa-miR-219-5p** | 2.6334 | 0.2336 | | | |
| **hsa-miR-137** | 2.6076 | 0.0119 | * | ** | |
| **hsa-miR-34a#** | 2.5690 | 0.0124 | * | ** | |
| **hsa-miR-1276** | 2.5607 | 0.5386 | | | |
| **hsa-miR-1254** | 2.5533 | 0.1846 | | | |
| **hsa-miR-34b** | 2.5217 | 0.3854 | | | |
| **hsa-miR-105#** | 2.4969 | 0.3282 | | | |
| **hsa-miR-181c#** | 2.4916 | 0.4524 | | | |
| **hsa-miR-30a-5p** | 2.4895 | 0.3112 | | | |
| **hsa-miR-541** | 2.4474 | 0.2580 | | | |
| **hsa-miR-34a** | 2.4435 | 0.0288 | * | ** | |
| **hsa-miR-24-1#** | 2.4386 | 0.4998 | | | |
| **hsa-miR-372** | 2.4304 | 0.2921 | | | |
| **hsa-miR-378** | 2.4265 | 0.1108 | | | |
| **hsa-miR-148a** | 2.3945 | 0.2100 | | | |
| **hsa-miR-302a** | 2.3877 | 0.5263 | | | |
| **hsa-miR-500** | 2.3729 | 0.5121 | | | |
| **hsa-miR-129#** | 2.3512 | 0.5045 | | | |
| **hsa-miR-1825** | 2.3082 | 0.2735 | | | |
| **hsa-miR-449b** | 2.3028 | 0.3194 | | | |
| **hsa-miR-1248** | 2.2801 | 0.2921 | | ** | |
| **hsa-miR-519d** | 2.2316 | 0.3407 | | | |
| **hsa-miR-23b** | 2.2083 | 0.3494 | | | |
| **hsa-miR-654-5p** | 2.1972 | 0.0883 | | | |
| **hsa-miR-99a#** | 2.1900 | 0.1765 | | | |
| **hsa-miR-148a#** | 2.1783 | 0.3760 | | | |
| **hsa-miR-486-5p** | 2.1572 | 0.3145 | | | |
| **hsa-miR-1274A** | 2.1451 | 0.0302 | * | ** | |
| **hsa-miR-1179** | 2.1444 | 0.0713 | | | |
| **hsa-miR-624** | 2.1427 | 0.3127 | | | |
| **hsa-miR-770-5p** | 2.1283 | 0.4983 | | | |
| **hsa-miR-7-2#** | 2.1159 | 0.3265 | | | |
| **hsa-miR-577** | 2.1074 | 0.3560 | | | |
| **hsa-miR-941** | 2.1024 | 0.5186 | | | |
| **hsa-miR-1270** | 0.4994 | 0.0679 | | | |
| **hsa-miR-25#** | 0.4978 | 0.1165 | | | |
| **hsa-miR-30d#** | 0.4891 | 0.0194 | * | | |

| | | | | | |
|---|---|---|---|---|---|
| **hsa-let-7a#** | 0.4817 | 0.0139 | * | | |
| **hsa-miR-661** | 0.4805 | 0.0458 | * | | |
| **hsa-miR-130b#** | 0.4723 | 0.0012 | ** | ** | |
| **hsa-miR-1324** | 0.4640 | 0.0561 | | | |
| **hsa-miR-888#** | 0.4516 | 0.2183 | | | |
| **hsa-miR-144** | 0.4341 | 0.1189 | | | |
| **hsa-miR-504** | 0.4282 | 0.0095 | ** | | |
| **has-miR-155** | 0.4198 | 0.0154 | * | | |
| **hsa-miR-10a** | 0.4088 | 0.0368 | * | | |
| **hsa-miR-182** | 0.3945 | 0.0364 | * | | |
| **hsa-miR-16-1#** | 0.3906 | 0.0010 | *** | ** | |
| **hsa-miR-603** | 0.3878 | 0.1188 | | | |
| **hsa-miR-516-3p** | 0.3803 | 0.0466 | * | | |
| **hsa-miR-17#** | 0.3789 | 0.0337 | * | | |
| **hsa-miR-497#** | 0.3714 | 0.1172 | | | |
| **hsa-miR-132#** | 0.3705 | 0.0603 | | | |
| **hsa-miR-548K** | 0.3618 | 0.1168 | | | |
| **hsa-miR-634** | 0.3508 | 0.1162 | | | |
| **hsa-miR-219-1-3p** | 0.3367 | 0.0034 | ** | | |
| **hsa-miR-15a#** | 0.3341 | 0.0000 | *** | *** | |
| **hsa-miR-524** | 0.3222 | 0.0447 | * | | |
| **hsa-miR-630** | 0.3155 | 0.0655 | | | |
| **hsa-miR-15b#** | 0.2943 | 0.0051 | ** | | |
| **hsa-miR-597** | 0.2913 | 0.0033 | ** | | |
| **hsa-miR-1183** | 0.2834 | 0.0042 | ** | | |
| **hsa-miR-101#** | 0.2700 | 0.0000 | *** | *** | |
| **hsa-miR-144#** | 0.2635 | 0.0000 | *** | ** | |
| **hsa-miR-580** | 0.2552 | 0.0413 | * | | |
| **hsa-miR-1289** | 0.2510 | 0.0361 | | | |
| **hsa-miR-600** | 0.2414 | 0.0062 | ** | | |
| **hsa-miR-483-3p** | 0.2311 | 0.0024 | ** | | |
| **hsa-miR-643** | 0.2264 | 0.0005 | *** | ** | |
| **hsa-miR-548d-3p** | 0.1953 | 0.0014 | ** | | |
| **hsa-miR-566** | 0.1685 | 0.0000 | *** | ** | |
| **hsa-miR-135b#** | 0.1651 | 0.0005 | *** | | |
| **hsa-miR-644** | 0.1324 | 0.0010 | *** | | |
| **hsa-miR-1278** | 0.0956 | 0.0000 | *** | ** | |
| **hsa-miR-23a#** | 0.0791 | 0.0003 | *** | | |
| **hsa-miR-1285** | 0.0759 | 0.0001 | *** | | |

miRNA that were either up-regulated or down-regulated by 2-fold in cisplatin induced senescent fibroblasts. The data represents fold change calculated from $\Delta\Delta C_t$ values normalized to U6 endogenous control and proliferating control. *p<0.05, by multiple paired t-test with and without Holm-Sidak corrections and using false discovery rate (FDR) set to 0.3.

**Table S2. Top 50 miRNA interacting pathways in senescent oral fibroblasts.** The DIANA-miRPath tool was used to predict the genes and putative pathways that may be deregulated in senescent oral fibroblasts.

| KEGG pathway | p-value | #genes | #miRNAs |
|---|---|---|---|
| **PI3K-Akt signaling pathway** | 1.24E-35 | 158 | 28 |
| **Pathways in cancer** | 1.93E-36 | 165 | 28 |
| **Focal adhesion** | 9.74E-33 | 100 | 28 |
| **Neurotrophin signaling pathway** | 3.88E-28 | 70 | 28 |
| **HTLV-I infection** | 4.87E-16 | 117 | 28 |
| **Chemokine signaling pathway** | 2.28E-07 | 77 | 28 |
| **Tight junction** | 1.54E-05 | 59 | 28 |
| **MAPK signaling pathway** | 6.28E-36 | 127 | 27 |
| **Melanogenesis** | 1.36E-06 | 45 | 27 |
| **Transcriptional misregulation in cancer** | 3.66E-06 | 80 | 27 |
| **Viral carcinogenesis** | 0.002974748 | 80 | 27 |
| **Protein processing in endoplasmic reticulum** | 0.007563723 | 67 | 27 |
| **Wnt signaling pathway** | 1.92E-28 | 81 | 26 |
| **Hepatitis B** | 9.59E-27 | 76 | 26 |
| **Ubiquitin mediated proteolysis** | 5.83E-20 | 68 | 26 |
| **Axon guidance** | 8.59E-20 | 69 | 26 |
| **Renal cell carcinoma** | 1.31E-19 | 42 | 26 |
| **Dopaminergic synapse** | 2.01E-16 | 64 | 26 |
| **Endometrial cancer** | 2.83E-13 | 31 | 26 |
| **Glutamatergic synapse** | 2.06E-05 | 53 | 26 |
| **Epstein-Barr virus infection** | 0.000155449 | 82 | 26 |
| **Tuberculosis** | 0.01557663 | 66 | 26 |
| **Herpes simplex infection** | 0.04133367 | 70 | 26 |
| **Prostate cancer** | 5.54E-23 | 57 | 25 |
| **Colorectal cancer** | 1.13E-17 | 41 | 25 |
| **Gap junction** | 1.10E-14 | 45 | 25 |
| **Regulation of actin cytoskeleton** | 4.70E-10 | 93 | 25 |
| **Retrograde endocannabinoid signaling** | 3.30E-09 | 53 | 25 |
| **T cell receptor signaling pathway** | 1.24E-07 | 48 | 25 |
| **Influenza A** | 4.03E-05 | 69 | 25 |
| **RNA transport** | 0.000593956 | 61 | 25 |
| **Oocyte meiosis** | 0.000955549 | 51 | 25 |
| **ErbB signaling pathway** | 1.09E-23 | 55 | 24 |
| **Insulin signaling pathway** | 1.39E-23 | 68 | 24 |
| **Glioma** | 4.22E-16 | 41 | 24 |
| **HIF-1 signaling pathway** | 6.83E-15 | 54 | 24 |
| **Long-term potentiation** | 1.92E-14 | 36 | 24 |
| **GnRH signaling pathway** | 4.03E-13 | 44 | 24 |
| **mRNA surveillance pathway** | 1.24E-10 | 43 | 24 |
| **Measles** | 4.36E-09 | 64 | 24 |
| **Amoebiasis** | 4.52E-09 | 47 | 24 |
| **B cell receptor signaling pathway** | 6.88E-09 | 36 | 24 |
| **Cholinergic synapse** | 9.83E-07 | 52 | 24 |
| **Serotonergic synapse** | 0.00618267 | 44 | 24 |
| **Endocytosis** | 8.30E-32 | 102 | 23 |
| **Pancreatic cancer** | 1.69E-20 | 46 | 23 |
| **Melanoma** | 5.81E-16 | 38 | 23 |
| **mTOR signaling pathway** | 1.15E-13 | 34 | 23 |
| **Osteoclast differentiation** | 5.88E-12 | 60 | 23 |
| **Basal cell carcinoma** | 3.74E-09 | 27 | 23 |

**Table S3. Seed sequences of differentially expressed miRNA in senescent oral fibroblasts predicted to target the PTEN 3'UTR.** The seed sequences were obtained by *in silico* analysis using targetscan.org, version 6.2.

<table>
<tr><th>miRNA</th><th>Region and seed sequence</th></tr>
<tr><td>miR-137</td><td>4005-4011 5' UGCAAUAAU 3'<br>||||||<br>miR-137 3' UCGUUAUU 5'</td></tr>
<tr><td>miR-148b</td><td>2254-2260 5'UUUUGCACUGU 3'<br>| |||||||||<br>miR-148b 3'ACUACGUGACU 5'<br><br>3151-3158 5'UAUGCACUGA 3'<br>||||||||||<br>miR-148b 3'CUACGUGACU 5'</td></tr>
<tr><td>miR-29b</td><td>676-683 5' UUGGUGCUA 3'<br>|||||||<br>miR-29b-3p 3' UACCACGAU 5'<br><br>1741-1747 5' UUGGUGCUG 3'<br>|||||||<br>miR-29b-3p 3' UACCACGAU 5'</td></tr>
<tr><td>miR-200a</td><td>1467-1473 5' GCAGUGUUG 3'<br>|||||||<br>miR-200a-3p 3' -GUCACAAU 5'<br><br>3257-3263 5' UAGUGUUAU 3'<br>||||||<br>miR-200a-3p 3' GUCACAAU 5'<br><br>4252-4258 5' CCAGUGUUU 3'<br>|||||||<br>miR-200a-3p 3' UGUCACAAU 5'</td></tr>
<tr><td>miR-519a</td><td>413-419 5' UUGCACUUG 3'<br>|||||||<br>miR-519a-3p 3' UACGUGAAA 5'<br><br>1148-1155 5' AUGCACUUA 3'<br>|||||||<br>miR-519a-3p 3' UACGUGAAA 5'</td></tr>
</table>
